# Effects of the COVID-19 pandemic on the activity of clinical laboratories in Spain, evolution in the 2019–2021 period

**DOI:** 10.1515/almed-2022-0107

**Published:** 2022-12-06

**Authors:** Ana Belén Lasierra Monclús, Álvaro González, Francisco A. Bernabéu Andreu, Imma Caballé Martín, Antonio Buño Soto, Mercè Ibarz, Concepción González Rodríguez, José Puzo Foncillas

**Affiliations:** Service of Clinical Analysis and Biochemistry, Hospital Universitario San Jorge, Huesca, Spain; Service of Biochemistry, Clínica Universidad de Navarra, Pamplona, Spain; Service of Biochemistry-Clinical Analysis, Hospital Universitario Puerta de Hierro, Majadahonda, Spain; CATLAB Central Laboratory, Barcelona, Spain; Service of Clinical Analysis, Hospital Universitario La Paz, Madrid, Spain; Service of Clinical Analysis, Hospital Universitario Arnau de Vilanova, Lleida, Spain; Service of Biochemistry-Clinical Analysis, Hospital Universitario Virgen Macarena, Sevilla, Spain

**Keywords:** chronic disease, clinical laboratories, COVID-19, healthcare activity, population programs

## Abstract

**Objectives:**

To assess the impact of the COVID-19 pandemic on the activity of clinical laboratories in Spain.

**Methods:**

A descriptive, observational, retrospective, multicenter study.

**Results:**

Between March and December 2020, there was a statistically significant decrease in the number of test requests (−17.7%, p=<0.001) and total tests performed (−18.3%, p<0.001) with respect to the same period in 2019. A decrease was observed in the number of requests from primary care (−37.4%) (p<0.001) and in the number of foecal occult blood (−45.8%); qualitative urine (−30.1%); PSA (−28.5%); TSH (−27.8%); total cholesterol (−27.2%) and HbA_1c_ (−24.7%) tests performed, p<0.001. A significant increase was found in the number of requests from ICUs (76.6%, p<0.001) and number of IL-6 (+22,350.9), D-dimer (+617.2%), troponin (+46.8%) and arterial blood gas (+3.9%) tests carried out, p<0.001. During the first months of 2021, there were significant changes in the number of requests for qualitative urine (−8.7%, p<0.001), PSA (−6.3%, p=0.009), IL-6 (+66,269.2, p<0.001), D-dimer (+603.6%, p<0.001), troponin (+28.7%, p<0.001), arterial blood gas (+26,2%, p=0.014) and ferritin (+16.0%, p=0.002) tests performed.

**Conclusions:**

There were changes in the origin and number of test requested to clinical laboratories in Spain. The number of requests for the evaluation and monitoring of COVID-19 patients increased, whereas requests for the control of non-COVID patients and for population screening decreased. Long-term analysis reveals that the volume of tests performed for the control of chronic diseases returned to normal over time, whereas the increase observed in the volume of tests performed for the management of COVID-19 patients is maintained.

## Introduction

In December 31, 2019, a case series of pneumonia of unknown origin was reported in Wuhan (province of Hubei, China) [1]. In January 2020, the Chinese health authorities identified a novel coronavirus as the causative agent of these cases of atypical pneumonia, and published the genetic sequence of the causative agent. The novel coronavirus was named 2019-nCoV (from English: *2019-novel coronavirus*) [2]. After the first case outside the People’s Republic of China was officially confirmed by the World Health Organization (WHO) on January 13, 2020, the number of infected patients increased dramatically, and the first deaths were reported. On February 11, 2020, the WHO named the disease caused by the novel coronavirus as COVID-19, which is the abbreviated form of *coronavirus disease 2019* [1]. The causative agent was called SARS-CoV-2 by the International Committee on Taxonomy of Viruses [3]. On March 11, the WHO declared COVID-19 a pandemic due to the alarming levels of spread and severity, and the lack of action. At that moment, Europe had become the epicenter of the pandemic, with the highest number of reported cases and deaths as compared to the rest of regions of the world together, apart from the People’s Republic of China [1]. The first case of COVID-19 reported in Spain was diagnosed on January 31, 2020 in La Gomera (Canary Islands). The second case was diagnosed on February 9, 2020 in Palma de Mallorca (Balearic Islands). The two cases were mild and imported, and had had contact with a confirmed case of SARS-COV-2 infection in Germany and France, respectively [4]. The first cases of COVID-19 in the peninsula were reported by the end of February 2020, a total of 24 [5], whereas as per March 13, 2020, the number of probable cases reported in Spain was 4,209 [6]. On March 14, 2020, the state of alarm was declared in Spain, which involved a lockdown of the population to stop the spread of the pandemic [7]. Due to the strict measures adopted, healthcare activity was restricted to diseases that required urgent management. Consultations and elective surgical procedures were cancelled to avoid unnecessary interactions and contacts [8]. Some studies show that even urgent activity decreased in some specialties [9].

The COVID-19 pandemic had a dramatic impact on healthcare activity. Activity in healthcare Services, either in primary and secondary care, was crucial in the battle against the disease. Clinical laboratories are recognized to have played a key role in the diagnosis, evaluation and management of COVID-19 [10]. Clinical laboratories in Spain had to rapidly adapt to these changes in the demand for tests aimed at determining the severity of COVID-19 patients, predict its course and perform therapeutic monitoring. During the first waves, the rapid growth of cases and hospital admissions of patients with severe COVID-19 resulted in a substantial increase in the demand for laboratory tests. New tests had to be rapidly developed. Laboratories had to cope with the shortage of reagents and supplies and the unavailability of personnel due to COVID-19 infections.

In addition, the lockdown and the COVID-19 itself may have had a negative impact on the management of other diseases. An example is the poorer control of chronic diseases [11], [12], [13], which would be manifested in a decrease in the number of test requests for the monitoring of these diseases. It has been proven that this significant change in routine healthcare activity has caused an increase in obesity and other factors associated with the etiology of chronic diseases [14], [15], [16].

This study was aimed at assessing potential changes in the origin, complexity, and type of test requests received by clinical laboratories (Services of Clinical Analysis and Clinical Biochemistry) of hospitals in Spain. To such purpose, an analysis was conducted of the analytes used for the evaluation and monitoring of hospitalized COVID-19 patients. Additionally, an analysis was carried out of routine control tests performed in patients with chronic diseases such as diabetes and cancer, among others.

The purpose of this study is to determine the impact of the COVID-19 pandemic on the activity of clinical laboratories of hospitals in Spain.

## Materials and methods

A descriptive, observational, retrospective, multicenter study was conducted in seven Clinical Analysis and/or Clinical Biochemistry laboratories located in different provinces of Spain. Six of the laboratories are public, whereas one is private (laboratory 2). All serve patients from emergency departments, ICUs, hospitalization wards and outpatient units. Laboratory 2 is the only that does not serve primary care patients.

A database was created with monthly data for the study period (January 2019 -June 2021). Each laboratory collected retrospective monthly data for the following variables: total number of requests and number of requests by type of requesting service (Emergency Care, Hospitalization ward, ICU, Outpatient Consultations, and Primary Care), as well as the total of analyses requested and number of tests performed for glucose; creatinine; total cholesterol; aspartate tests aminotransferase (AST); ferritin; troponin; thyrotropin (TSH); prostate-specific antigen (PSA); carcinoembryonic antigen (CEA); foecal occult blood (FOB); glycated haemoglobin (HbA_1c_); interleukin 6 (IL-6); C-reactive protein (CRP); qualitative urinalysis; arterial blood gas; venous blood gas; and D-dimer. Analytical tests were selected based on their relevance in the management of patients with SARS-CoV-2 infection [17] and control of non-COVID patients (chronic diseases, colorectal cancer screening programs, among others). Microbiology requests or tests were not included, as they are out of the scope of this study.

Data were extracted for the laboratory information system (LIS) of each center. Personal data were not used. Approval from the Ethics Committee was not required, since data were extracted from the standard information systems of the participating clinical laboratories.

### Statistical analysis

Absolute and relative differences were calculated by taking the pre-pandemic period as a reference (January–December 2019). As data were not normally distributed on the Kolmogorov-Smirnov test, statistically significant differences were analyzed using the non-parametric Wilcoxon test for paired samples. To detect specific changes in each laboratory, the Wilcoxon test for paired samples was applied again in a segmented manner fo each of the laboratories.

The percentage of change with respect to the 2019 mean of each of the variables recorded for each of the laboratories was calculated and plotted, to objectively analyze changes throughout the study period.

All statistical tests were considered bilateral and statistically significant for p-values <0.05. Data were analyzed using the statistical SPSS 25.0 software (IBM Corporation, Armnok, NY, USA).

## Results

Table 1 contains general data for the 7 participating laboratories. Changes (absolute and relative differences) were observed in the number of requests and tests performed between March and December 2020 (pandemic period), as compared to the same period of 2019 (pre-pandemic period).

**Table 1:** Changes in the use of clinical laboratories (number of requests and tests performed) between the pandemic period (March–December 2020) and the same prepandemic period (March–December 2019).

	March–December 2020, n	March–December 2019, n	Absolute differences, n	Relative differences, %	p-Value^a^
**Total requests**	2,611,129	3,173,523	−562,394	−17.7	<0.001
Primary care requests	749,444	1,197,783	−448,339	−37.4	<0.001
Hospitalization ward requests	570,271	551,235	19,036	3.5	0.449
Outpatient unit requests	646,752	743,894	−97,142	−13.1	<0.001
Urgent requests	766,366	805,420	−39,054	−4.9	0.006
ICU requests	103,814	58,797	45,017	76.6	<0.001
**Total tests**	25,617,642	31,357,303	−5,739,661	−18.3	<0.001
Glucose	1,608,217	2,061,963	−453,746	−22.0	<0.001
Creatinine	1,432,234	1,782,129	−349,895	−19.6	<0.001
Total cholesterol	969,661	1,332,265	−362,604	−27.2	<0.001
AST	1,009,605	1,197,242	−187,637	−15.7	<0.001
C-reactive protein	730,604	805,277	−74,673	−9.3	0.107
Ferritin	477,800	513,024	−35,224	−6.9	0.318
IL-6	11,899	53	11,846	22,350.9	<0.001
Troponin	155,194	105,700	49,494	46.8	<0.001
TSH	558,758	773,631	−214,873	−27.8	<0.001
HbA_1c_	274,037	363,784	−89,747	−24.7	<0.001
CEA	63,646	73,014	−9,368	−12.8	0.007
PSA	124,914	174,656	−49,742	−28.5	<0.001
FOB	70,723	130,424	−59,701	−45.8	<0.001
Qualitative urine test	490,504	701,910	−211,406	−30.1	<0.001
Arterial blood gas	164,800	121,227	43,573	35.9	<0.001
Venous blood gas	193,295	195,137	−1,842	−0.9	0.560
D-dimer	170,312	23,748	146,564	617.2	<0.001

^a^Non-parametric Wilcoxon’s test for paired data (n=70). AST, aspartate aminotransferase; CEA, carcinoembryonic antigen; HbA_1c_, glycated haemoglobin; IL-6, interleukin 6; PSA, prostate-specific antigen; FOB, foecal occult blood; TSH, thyrotropin.

The results of this analysis reveal a statistically significant decline in clinical activity in laboratories in 2020 (−17.7% total requests, p=<0.001 and −18.3% total tests, p<0.001) in the March–December 2020 period, as compared to the same period of 2019. Thus, a substantial decrease of 37.4% was observed in the volume of requests from Primary Care, with respect to the same period of 2019 (p<0.001). In addition, there was an 18.30% reduction in the total of tests and, more specifically, in the number of tests performed for FOB (−45.8%); qualitative urine (−30.1%); PSA (−28.5%); TSH (−27.8%); total cholesterol (−27.2%); and HbA_1c_ (−24.7%), p<0.001 for all.

In addition, the number of test requests from ICUs showed a significant increase of 76.6% in 2020 with respect to 2019 (p<0.001). A considerable increase was found in the number of tests related to the management of COVID-19 patients, including IL-6 (+22,350.9%, which was not available in most laboratories until 2020); D-dimer (+617.2%), troponin (+46.8%) and arterial blood gas (+35.9%), p<0.001 for all.

Pooled data for all participating laboratories show that the decrease in the number of total requests with respect to the same period of 2019 was much more significant during the first wave of the pandemic (March–May 2020) (−35.7%, p<0.001). Hence, in this period, there was a substantial decrease in the number of requests from primary care (−63.9%, p<0.001) and outpatient units (−38.3%, p<0.001), and an increase in the volume of requests from ICUs (+100.5%, p=0.001). During this period, the number of total tests performed decreased by 40.6%, p<0.001.

To assess the impact of the COVID-19 pandemic in the long-term, activity in clinical laboratories during the January–June 2021 period was compared to the same period of 2019 (Table 2).

**Tabla 2:** Long-term changes in the use of clinical laboratories (number of requests and tests performed) between the pandemic period (January–June 2021) and the most recent prepandemic period (January–June 2019).

	January–June 2021, n	January–June 2019, n	Absolute differences, n	Relative differences, %	p-Value^a^
**Total requests**	2,001,270	1,965,293	35,977	1.8	0.126
Primary care requests	677,047	745,478	−68,431	−9.2	<0.001
Hospitalization ward requests	426,161	342,390	83,771	24.5	0.002
Outpatient unit requests	483,598	462,865	20,733	4.5	0.123
Urgent requests	501,461	488,654	12,807	2.6	0.778
ICU requests	51,392	35,084	16,308	46.5	<0.001
**Total tests**	19,797,233	19,615,906	181,327	0.9	0.330
Glucose	1,246,609	1,289,491	−42,882	−3.3	0.232
Creatinine	1,103,286	1,112,368	−9,082	−0.8	0.817
Total cholesterol	791,416	840,895	−49,479	−5.9	0.062
AST	765,502	763,097	2,405	0.3	0.253
C-reactive protein	519,752	511,334	8,418	1.7	0.022
Ferritin	378,728	326,457	52,271	16.0	0.002
IL-6	8,628	13	8,615	66,269.2	<0.001
Troponin	86,352	67,090	19,262	28.7	<0.001
TSH	488,524	485,763	2,761	0.6	0.232
HbA_1c_	246,167	224,906	21,261	9.5	<0.001
CEA	46,118	45,837	281	0.6	0.604
PSA	105,350	112,400	−7,050	−6.3	0.009
FOB	79,120	81,893	−2,773	−3.4	0.167
Qualitative urine test	394,364	431,893	−37,529	−8.7	<0.001
Arterial blood gas	105,844	83,890	21,954	26.2	0.014
Venous blood gas	132,314	115,937	16,377	14.1	<0.001
D-dimer	103,844	14,760	89,084	603.6	<0.001

^a^Non-parametric Wilcoxon’s test for paired data (n=70). AST, aspartate aminotransferase; CEA, carcinoembryonic antigen; HbA_1c_, glycated haemoglobin; IL-6, interleukin 6; PSA, prostate-specific antigen; FOB, foecal occult blood; TSH, thyrotropin.

Interestingly, during the 2021 period, the total of test requests was similar to that of 2019 (+1.8%, p=0.126). However, the type of requesting service changed, with an increase with respect to 2019 in the number of requests from ICUs (+46.5%, p<0.001) and hospitalization wards (+24.5%, p=0.002). Although less dramatic than in 2020, a decrease was also observed in 2021 in the number of requests from Primary Care (−9.2%, p<0.001). Likewise, the total of tests performed in 2021 increased with respect to 2020 and was similar to that of 2019 (+0.9%, p=0.330). The number of tests for FOB (−3.4%), qualitative urine (−8.7%) and PSA (−6.3%) increased with respect to 2020, but did not reach 2019 values. In 2021, an increase was observed in the number of HbA_1c_ tests with respect to 2019 (+9.5%, p<0.001).

The number of requests associated with the management of COVID-19 patients kept growing in 2021. There was an increase with respect to 2019 in the number of tests for IL-6 (+66,269.2, p<0.001); D-dimer (+603.6%, p<0.001); troponin (+28.7%, p<0.001); arterial blood gas (+26.2%, p=0.014) and ferritin (+16.0%, p=0.002), which was even more substantial for IL-6 and ferritin than in 2020.

**Figure 1:**
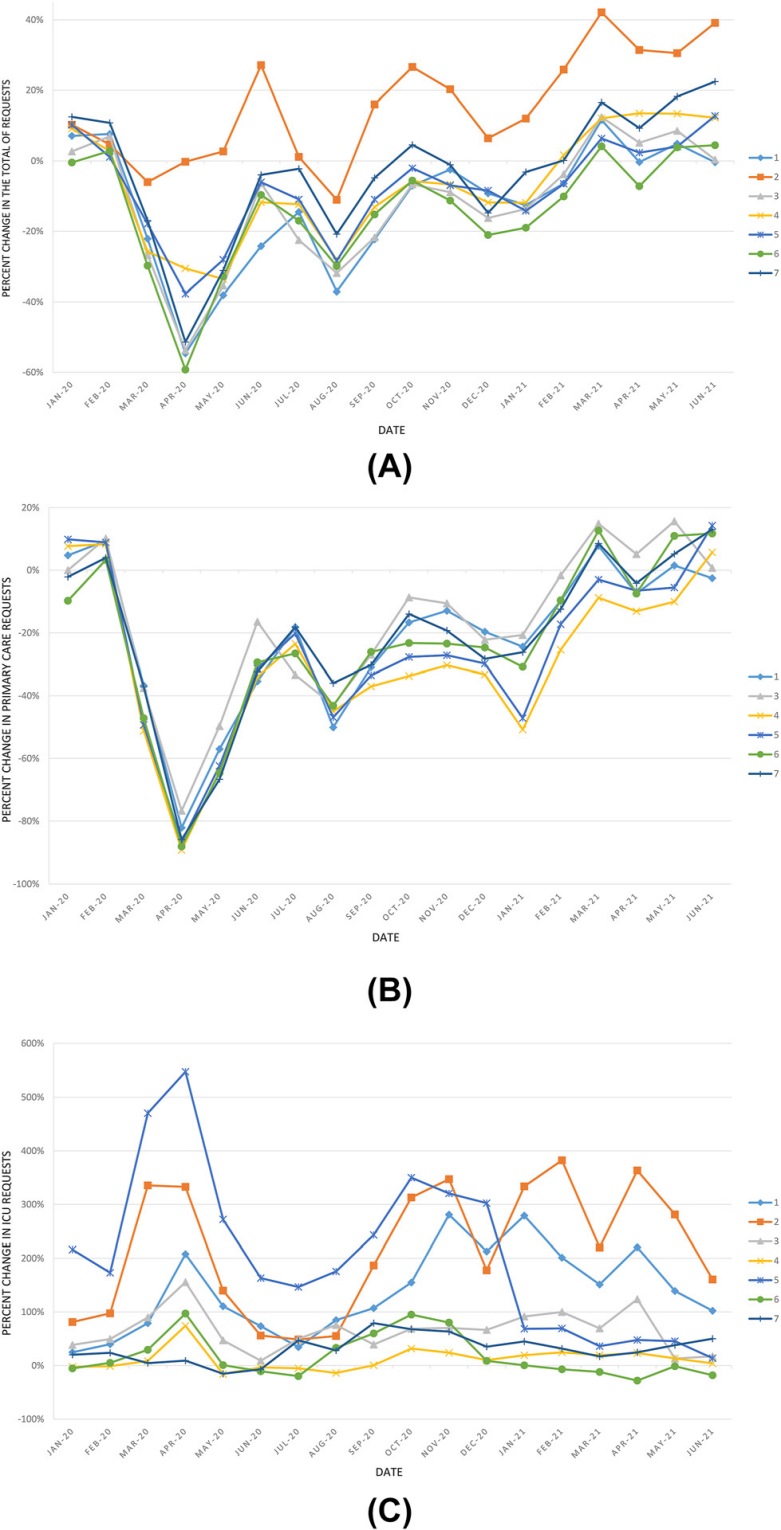
Percent change in the number of requests received in each of the 7 laboratories, with respect to their corresponding average in 2019 during the study period: (A) total of requests; (B) number of requests from primary care (Laboratory 2 is excluded, since it does not serve this type of patients) and (C) number of requests from the ICU.

**Figure 2:**
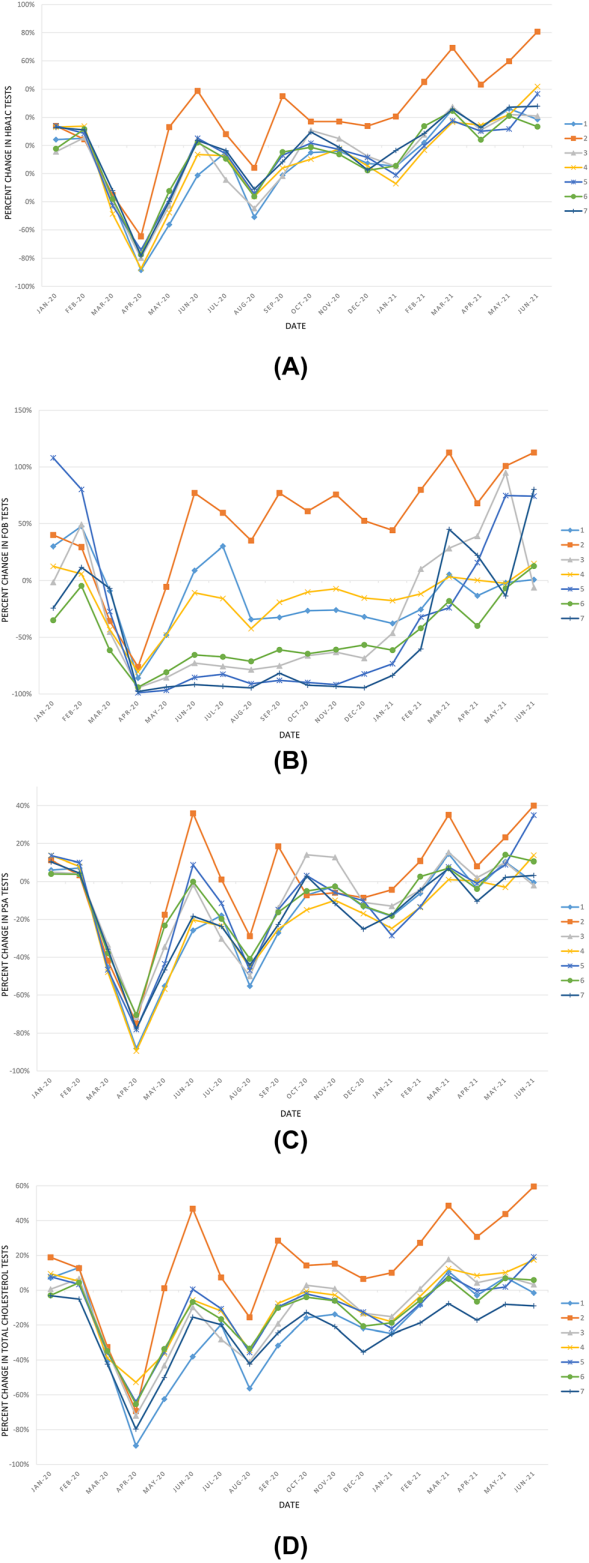
Percent change in the number of laboratory tests for the control of patients with diseases other than COVID-19 by each of the 7 laboratories, with respect to their corresponding average in 2019, during the study period. (A) HbA_1c_; (B) SOH; (C) PSA and (D) Total cholesterol. HbA_1c_, glycated haemoglobin; PSA, prostate specific antigen; FOB, foecal occult blood test.

**Figure 3:**
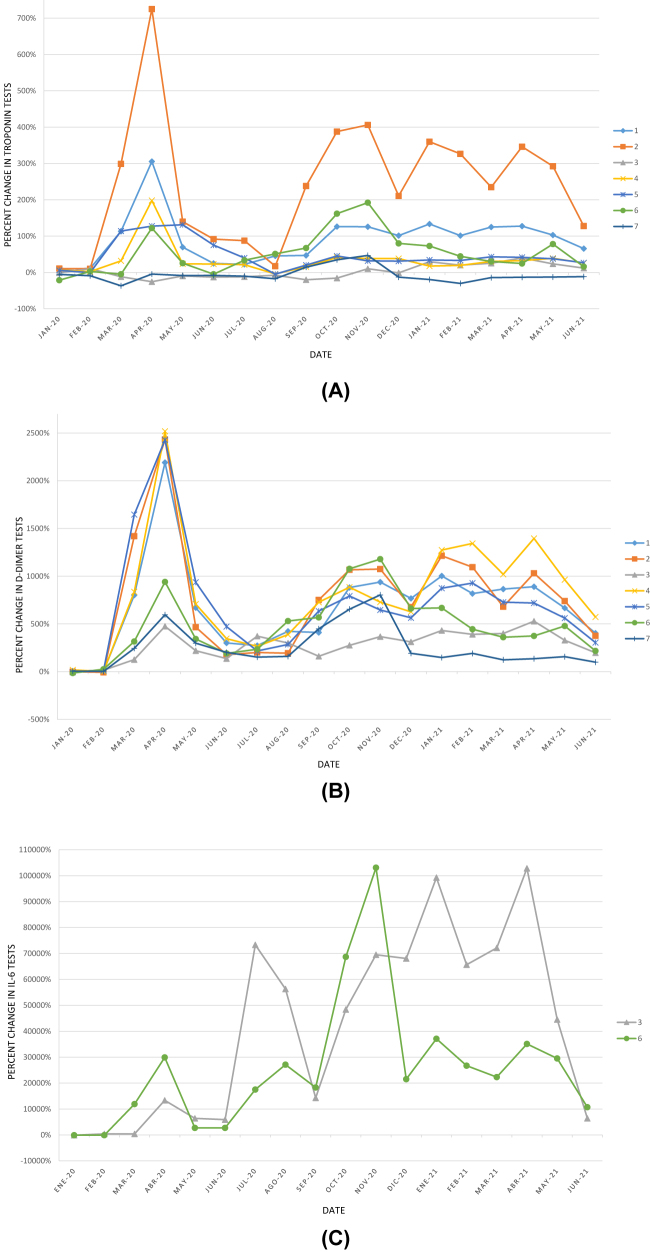
Percent change in the number of laboratory tests for the control and management of COVID-19 patients in each of the 7 laboratories, with respect to the average in 2019, during the study period. (A) troponin; (B) D-dimer; (C) IL-6 (data shown for the laboratories where this test was available in 2019). IL-6, interleukin 6.

The figures below show the percent change in the number of requests with respect to the 2019 mean by laboratory between January 2020 and June 2021 (Figure 1), and the percent change in the number of tests for several of the analytes studied (Figures 2 and 3).

## Discussion

The outbreak of the COVID-19 pandemic led numerous countries to declare the state of alarm in March 2020 to try to control the spread of the virus and avoid the collapse of healthcare systems. Spain was one of the countries most affected by the measures adopted by the administrations to control the pandemic, which included a home lockdown for the general population and the restriction of work activity to essential services.

This analysis of activity in clinical laboratories in Spain during the pandemic may help design strategies and improve the use of healthcare resources in future pandemics.

Most of the studies available in the literature on the use of clinical laboratories during the COVID-19 pandemic were focused on the diagnosis of SARS-COV-2 infection, where microbiology laboratories were essential [18]. Other studies evaluated tests for the diagnosis, monitoring of severity and prognosis of patients infected with SARS-CoV-2 [19, 20]. However, there is scarcity of data on the demand for tests related and unrelated to COVID-19 received by clinical laboratories [12, 13]. Virtually no studies have been conducted to assess the impact of the pandemic on the number of tests performed for analytes related to other chronic diseases [21], which are most commonly requested by primary care, and none has been carried out in Spain.

Durant et al. [12] reported a decrease in the volume of tests (−27.6%) performed in their central laboratory during the second and third week of March 2020, with respect to the same period of 2019. The decrease documented by Durant during the first wave was less significant that the one detected in our study (March–May 2020), with respect to the same period of 2019 (−40.6% total tests). Notably, the authors reported an increase in the number of tests for ferritin (+335.6%), whereas we did not observe any statistically significant differences in ferritin tests in our study (−6.9%, p=0.318). In contrast, consistently with our results, Durant et al. documented significant increases in tests for troponin (+235.6% vs. +46.8% in our study); D-dimer (+1,771.6% vs. +617.2%); and arterial blood gas (+24.1% vs. +35.9). Although the volume of ferritin test requests increased during the pandemic due to its association with inflammatory status in COVID-19 patients, this increase was not observed in our study due to the dramatic decrease in tests for anemia. It is worth mentioning that Duran et al. [12] conducted a descriptive but not a statistical analysis to objectively assess changes in the number of tests. Besides, they did not analyze other analytes related to the management of non-COVID-19 patients.

During the pandemic, attention to patients with chronic diseases deteriorated, due to restrictions to non-urgent elective consultations and patient’s fear of being exposed to the coronavirus during face-to-face visits [11]. Chronic diseases cause the death of 41 million every year, which accounts for 71% of all deaths worldwide. Cardiovascular diseases (17.9 million patients per year); cancer (9.3 millions); respiratory diseases (4.1 millions); and diabetes (1.5 millions) account for 80% of early deaths [22].

A study was undertaken in U.S.A. to assess the impact of COVID-19 on six chronic diseases (congestive heart failure, chronic obstructive pulmonary disease, type 2 diabetes, hypertension, chronic kidney disease and cancer), with a 50% reduction in *de novo* diagnoses for all these diseases, and 30–60% reductions in the number of face-to-face consultations. At the end of June 2020, the number of medical visits recovered and reached 70–80% of visits in the previous years. In contrast, non-medical visits (imaging studies and laboratory tests, among others) recovered only to 55% of visits in the previous years [13]. A higher increase was observed in our study, where the total of test requested in June 2020 reached 79.1% of the tests requested in the same period of 2019. Notably, this increase is less significant when only requests from primary care are considered (29.7% in our study).

In Spain, non-emergency activity decreased dramatically in primary care during the first wave (including laboratory tests, follow-up of chronic patients and population cancer screening tests), since priority was given to the management of COVID-19 patients [23]. The number of test requested from primary care decreased in the March–December 2020 period with respect to the same period in 2019, a decrease that was more considerable during the first wave (−63.9%). This result is consistent with that of Nagy et al. [21], who reported a decrease of 46–71% in the number of auto-antibody tests usually requested from primary care. Our results reveal a reduction in the number of tests for FOB, qualitative urine, PSA, TSH, total cholesterol and HbA_1c_ during the March–December 2020 period, with respect to the same period of 2019. This finding reveals a significant deterioration of the follow-up and control of non-COVID patients (diabetes, thyroid diseases, dyslipidemia, etc.), and colorectal cancer screening programs available in primary care.

Long-term follow-up (until June 2021) was performed to assess the situation of clinical laboratories more than a year after the outbreak of the pandemic. Although the number of requests from primary care during the first six months of 2021 decreased significantly with respect to the same period in 2019 (−9.2%, p<0.001), no significant differences were observed in most of the analytes studied (Table 2). These results suggest that, in 2021, the level of control of chronic diseases returned to normal, as compared to the significant decline observed in 2020, especially during the first wave of the pandemic. The number of test requests increased progressively in all participating laboratories (Figure 2). Of special mention is the dramatic decrease found in requests for FOB tests from the start of the pandemic (virtually −100%), which remained the same throughout 2020 in several of the hospitals included, and which recovery differed significantly as a function of the laboratory (Figure 2C). These data indicate that colorectal cancer screening programs were resumed later in some provinces or medical areas, with the corresponding loss of *de novo* cases detected in 2020.

In addition, the results show an increase in the number of tests for biomarkers used for the evaluation and monitoring of COVID-19 inpatients (IL-6, D-dimer, troponin, arterial blood gases and ferritin), which even increased in 2021 with respect to 2020 for IL-6 and ferritin. From the start of the pandemic, the understanding of the role of IL-6 in the management and treatment of COVID-19 patients improved progressively [17, 24], [25], [26], [27]. IL-6 testing became common practice in 2020 as an early marker of severe inflammatory response, COVID-19 severity [17, 24, 25], and as a criterion for the initiation of IL-6 inhibitor therapy [26, 27]. Before the pandemic, the IL-6 test was only available in three of the 7 participating laboratories. However, this marker was progressively included in the portfolio of services of clinical laboratories throughout 2020. In relation to ferritin tests, used both in basic screening for anemia and monitoring of COVID-19 patients, a significant increase was not observed in their number of requests in 2020. This may be explained by the fact that anemia studies decreased dramatically. In contrast, the volume of ferritin tests increased in 2021, since the number of control requests and tests were resumed in non-COVID-19 patients and remained elevated the requests in hospitalized and critically-ill COVID-19 patients.

A graphical analysis was performed of the evolution of activity in Clinical Analysis and/or Clinical Biochemistry laboratories over the 30-month study period (Figures 1–3). This analysis revealed that the number of requests and tests performed coincide with the waves of the COVID-19 pandemic (first wave: March–June 2020; second wave: July–December 2020; third wave: December 2020–March 2021; fourth wave: March–June 2021 [28]). The same peaks are observed in the volume of ICU requests (Figure 1C) and number of analytes associated with COVID-19 patient control (Figure 3). In contrast, inverse peaks are found in the total of requests and requests from primary care (Figure 1A, B) and the number of tests for analytes used in the management of non-COVID patients (Figure 2). There were differences in the impact of the different waves on the volume of requests in each clinical laboratory. This may be due to differences in the incidence of the pandemic across the different regions of the country, and to healthcare organizational issues that are out of the scope of this study (Figures 1C and 3).

The results of this study reveal that during 2020, Clinical Analysis and/or Clinical Biochemistry laboratories in Spain underwent a significant change in the origin of their requests and in the demand for tests by clinicians. Clinical laboratories had to rapidly adapt to these changes, reorganize their resources, create specific profiles for COVID-19 patients and incorporate new tests (i.e., IL-6) [17, 24, 25, 29]. In future public health crises, these findings may help optimize resource management and allocation. Thus, strategic plans in the future should provide for the use of material and human resources in clinical laboratories of a hospital or even between laboratories in different regions. Clinical laboratories should anticipate and coordinate to rapidly respond to the changing demand. Our study provides a general picture of changes in the activity of clinical laboratories, which may be useful for operational decision-making.

This study has some limitations. Firstly, although it was a multicenter study that included seven clinical laboratories from different regions, the sample may not be representative of all provinces. Secondly, organizational healthcare issues potentially associated with a risk of bias were not considered in this study. Finally, differences across laboratories were not analyzed. However, it is worth noting that a statistical analysis was performed to verify that the results obtained were representative of the seven participating laboratories.

In conclusion, clinical laboratories in Spain progressively adapted their activity to the requirements and needs that arouse as the waves of the pandemic surged and ebbed. To the best of our knowledge, this is the first study to analyze these data in Spain. In addition, this study assesses the demand for laboratory tests for the control and management of diseases other than COVID-19. From March 2020, the volume of requests for testing analytes associated with the evaluation and monitoring of hospitalized COVID-19 patients increased rapidly. In addition, the results obtained confirm a reduction in the volume of tests requested for the control of prevalent chronic diseases. As observed from the very beginning of the pandemic, diabetes, hypertension and obesity are risk factors that increase COVID-19 morbimortality [15, 16, 30]. In the coming years, we will observe the effects of the decrease in the control and management of chronic diseases and the reduction of cancer screening tests occurred in 2020, and their impact on morbimortality and future demand for clinical laboratory services.
